# Identification of a putative haplotype associated with recumbency in Holstein calves

**DOI:** 10.3168/jdsc.2022-0224

**Published:** 2022-08-06

**Authors:** C.D. Dechow, E. Frye, F.P. Maunsell

**Affiliations:** 1 Department of Animal Science, Pennsylvania State University, University Park 16802; 2 Department of Population Medicine, College of Veterinary Medicine, Cornell University, Ithaca, NY; 3 Department of Large Animal Clinical Sciences, College of Veterinary Medicine, University of Florida, Gainesville

## Abstract

Thirty-four Holstein calves from multiple farms were found recumbent during the neonatal period with no detectable neurologic, infectious, or metabolic abnormalities. Most calves did not survive beyond 6 wk of age. The objective of this study was to conduct a genome-wide association and pedigree analysis to determine if a genetic origin was plausible. There were 101,917 DNA markers for 18 affected calves and 26 unaffected family controls available for analysis. Genome-wide association, homozygosity screening, and a parental based transmission disequilibrium test were conducted in PLINK. A genomic region on the end of chromosome 16 that contained 78 markers based on a recessive inheritance model and that spanned 5.1 million bp was considered the most probable region for a genetic defect; the region was narrowed to 2.1 million bp following homozygosity screening and the transmission disequilibrium test with all affected calves homozygous in the candidate region and 1 homozygous control. A genotyped sire and 2 dams with imputed genotypes were heterozygous in the candidate region. A common sire born in 2008 was identified that was present for both paternal and maternal lineages of all affected calves; nearly all lineages traced through a prolific son born in 2010 who was genotyped and was heterozygous for the candidate region. Therefore, a possible genetic defect with incomplete penetrance on chromosome 16 that results in recumbency has been identified. Further efforts with an increase in families represented are needed to confirm a genetic basis, and identify the mutation and mode of inheritance.

## Highlights

• Thirty-four calves on 4 farms were unable to stand without assistance with most calves not surviving beyond 6 weeks of age.
• An incompletely penetrant haplotype on chromosome 16 was identified as a possible cause of the recumbent phenotype.
• Additional studies are required to confirm the genetic origin of the condition and identify the causative mutation.

## Graphical Abstract

**Figure:**
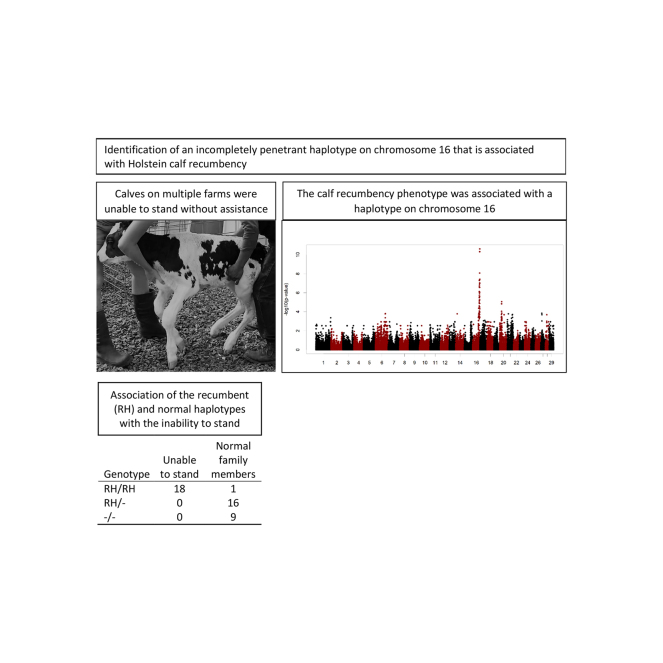
**Summary:** Thirty-four Holstein calves from 4 farms were found recumbent during the neonatal period. Most calves did not survive beyond 6 weeks of age, but others recovered. A genome-wide association and pedigree analysis was performed with 101,917 DNA markers for 18 affected calves and 26 unaffected family controls available. A 2.1 million bp genomic region on the end of chromosome 16 was considered the most probable region for a genetic defect. All affected calves were homozygous in the candidate region, as was one control calf. A common sire born in 2008 was identified that was present for both paternal and maternal lineages of all affected calves. Therefore, a possible genetic defect with incomplete penetrance on chromosome 16 that results in recumbency has been identified. Further efforts with an increase in families represented are needed to confirm a genetic basis, and identify the mutation and mode of inheritance.

Holstein genomic selection programs screen for several genetic recessives associated with stillbirth and late-term abortions (Agerholm et al., 2001; Charlier et al., 2012) and with neonate survival (Shuster et al., 1992; Schütz et al., 2016). Genomic testing has also accelerated discovery of genetic recessives that result in embryonic loss (Fritz et al., 2013, 2018; McClure et al., 2014; Adams et al., 2016) by screening for regions where a given haplotype is not observed in a homozygous state when expected to be present based on haplotype frequency (VanRaden et al., 2011b).

While genomic testing has facilitated rapid genetic improvement in addition to identification of genetic recessives, there has also been an acceleration of inbreeding when viewed on an annual and on a generational basis (Makanjuola et al., 2020). The current rise in inbreeding follows a period of contraction in male lineages due to the widespread adoption of AI (Dechow et al., 2020). Elevated inbreeding and small effective population sizes have increased the likelihood that new genetic recessive conditions will manifest in a significant number of animals (Fritz et al., 2013). Recently, recumbency despite normal mentation and appetite in newborn calves has been observed by veterinarians and farm staff in multiple states. Genetic defects are a prime contributor to neuromuscular disorders in humans and other species (Laing, 2012), and could underlie the problem observed in calves. Additionally, a neuropathy of genetic origin has been recently identified in Jersey cattle (Al-Khudhair et al., 2022). Therefore, the objectives of this study were to conduct a genome-wide association study of affected calves and normal family members to determine if a genetic origin was likely and to establish plausibility of genetic inheritance based on shared ancestors.

Table 1 provides details on the number of affected calves and the genotyped control population. Affected calves (34 total) were recorded on farms from New York state (farms 1 and 2), Florida (farm 3), and Pennsylvania (farm 4). The condition was first noted in December of 2019 with the latest observations from November of 2021. The affected calves were all weak and unable to stand without assistance at one or more times during the neonatal period with varying severity, recovery, and relapse patterns. Some calves were unable to stand after birth, whereas others were able to stand initially and then lost the ability to do so within the first 6 wk of life. Some recumbent calves regained the ability to stand after days to weeks, whereas others did not. Twenty-one calves were euthanized, 7 calves were considered recovered, and 6 were unthrifty with poor growth at the time of data collection. Because this was a retrospective study of affected calves, records and calf disposal were conducted by farm personnel per normal management protocols and not subject to an animal care and use protocol.

**Table 1:** The number of calves with recumbency and recovery status by farm, number of recumbent calves that resulted from embryo transfer, number of genotyped recumbent calves, number of genotyped control siblings, and number of parents or other relatives with genotypes^1^

Item	Farm 1	Farm 2	Farm 3	Farm 4
Recumbent (n)	10	3	17	4
Recovered (n)	1	0	5	1
Unhealthy (n)	1	0	5	0
Died or euthanized (n)	8	3	7	3
Sire × dam combinations (n)	10	2	3	2
Embryo transfer (n)	0	3	16	3
Recumbent calf genotypes (n)	1	1	12	4
Control sibling genotypes (n)	0	0	22	0
Parent genotypes (n)	0	0	3	0
Other relative genotypes (n)	0	0	0	1
Control birth weight (μ, kg)	N/A	N/A	35.7	N/A
Recumbent birth weight (μ, kg)	N/A	N/A	33.3	N/A

1 N/A = not available; μ = mean.

Calves were from 17 sire × dam combinations and 22 calves resulted from in vitro fertilization with full-sibling families of 13 (farm 3), 3 (farm 3 and 4), and 2 (farm 2). All farm 3 calves were sired by a single bull mated to 3 dams.

Blood samples were collected from 6 affected calves on farm 1 to determine if cholesterol deficiency, anemia, metabolic disorders, or selenium deficiency might contribute to recumbency. Of those 6 calves, one was necropsied on the farm by the attending veterinarian, with samples submitted to the Animal Health Diagnostic Center (**AHDC**), Cornell University College of Veterinary Medicine, and one calf was submitted directly to the AHDC for a full necropsy. One calf from farm 2 was necropsied by the attending veterinarian and samples were submitted to the AHDC. Blood samples were collected on farms 3 and 4 to evaluate selenium, and vitamin A, D, and E deficiency. The attending veterinarian for farm 3 performed 4 necropsies on farm, and 4 additional calves were submitted to the University of Florida College of Veterinary Medicine Veterinary Diagnostic Laboratories for full necropsies.

The behavior, mentation, and appetite of all calves was normal despite an inability to rise or remain standing. There was no evidence of metabolic abnormalities, anemia, cholesterol, selenium, or vitamin deficiency based on serum testing results. The necropsied calves from farm 1 were negative for neospora and bovine viral diarrhea (**BVD**) and the calf from farm 2 was negative for BVD, rabies, and listeria. All affected calves on farm 3 were negative for persistent infection with BVD. Gross necropsies failed to determine a cause for recumbency, and all examined histologic sections of the central nervous system, peripheral nerves, and muscle tissues were unremarkable. Recumbent calves weighed less at birth (mean = 33.3 kg; range = 26.4 to 38.6) than control siblings (mean = 35.7; range = 26.8 to 43.2) on farm 3 (Table 1), which was the only herd that recorded birth weight.

Tissue, hair, or blood samples were available for genotyping from 18 affected calves ranging from 1 to 12 per farm, with the remaining calves destroyed before sample collection could occur. For a control population, 22 healthy full siblings from 2 (17 and 5 calves, respectively) farm 3 dams were sampled. Additionally, the sire of the farm 3 calves and a full sister to the dam of 3 farm 4 calves were sampled. All samples were genotyped for 139,376 DNA markers (BOVUHDV03, Neogen). The 139,376 marker genotypes of 2 farm 3 dams were imputed with findhap.f90 (v3; Vanraden et al., 2011a) based on 8 and 26 genotyped offspring plus mate genotype and were included in subsequent analyses.

Recumbency was recorded as 1 for 26 unaffected animals including the control calves and genotyped relatives, or 2 for the 18 affected calves. Association analysis was conducted in PLINK (v1.9; Chang et al., 2015; www.cog-genomics.org/plink/1.9/). A marker call rate of ≥99% was imposed, leaving 101,907 makers for analysis. The initial association analysis was comprised of an allelic chi-squared test (frequency of allele A versus frequency of allele B) assuming 1 df with *P*-values generated using Fisher's exact test. This was followed with a recessive inheritance model (genotype AA and AB versus BB) with a 1 df chi-squared test. Multiple comparison testing was considered by implementing a false discovery rate (**FDR**) *P*-value to declare genome-wide significance at FDR *P* ≤ 0.05. Homozygosity screening was also performed with the number of affected and normal animals homozygous for each marker determined. Finally, a transmission disequilibrium test (**TDT**) was conducted for 12 affected calves that had genotyped parents from farm 3.

A Manhattan plot demonstrating the significance level for each marker according to the recessive inheritance model is shown in Figure 1 with a peak apparent at the end of chromosome 16. In total, 78 markers in the region were significant (FDR *P* ≤ 0.05) over a 5.1 million bp region that ranged from 75,332,437 to 80,448,457 bp (ARS-UCD 1.2). The allelic model returned similar results with more significant markers (88) over a slightly expanded range (5.7 million bp). The affected calves had a shared run of homozygosity that spanned 2.1 million bp from 78,732,954 to 80,748,266; one control calf was homozygous over the same region and there were no other regions where all 18 affected genotyped calves shared homozygosity. For the TDT model, markers spanning from 78,936,848 to 80,4484,57 tended toward genome-wide significance (FDR *P* = 0.09) and were significant (FDR *P* = 0.003) on a chromosome-wide basis. The association of a 120-marker haplotype ranging from 78,732,954 to 80,748,266 bp with the recumbency phenotype is shown in Table 2. All affected calves were homozygous and 1 of 26 controls was homozygous from farm 3; 9 controls were homozygous for the alternate haplotype and 16 were heterozygous. The sire of the farm 3 calves and both dams were heterozygous for markers in the region. A chi-squared test using the FREQ procedure in SAS (v 9.4, SAS Institute Inc.) was applied to the results in Table 2 with the null hypothesis of no association between genotype and recumbency rejected (*P* < 0.0001).

**Figure 1:**
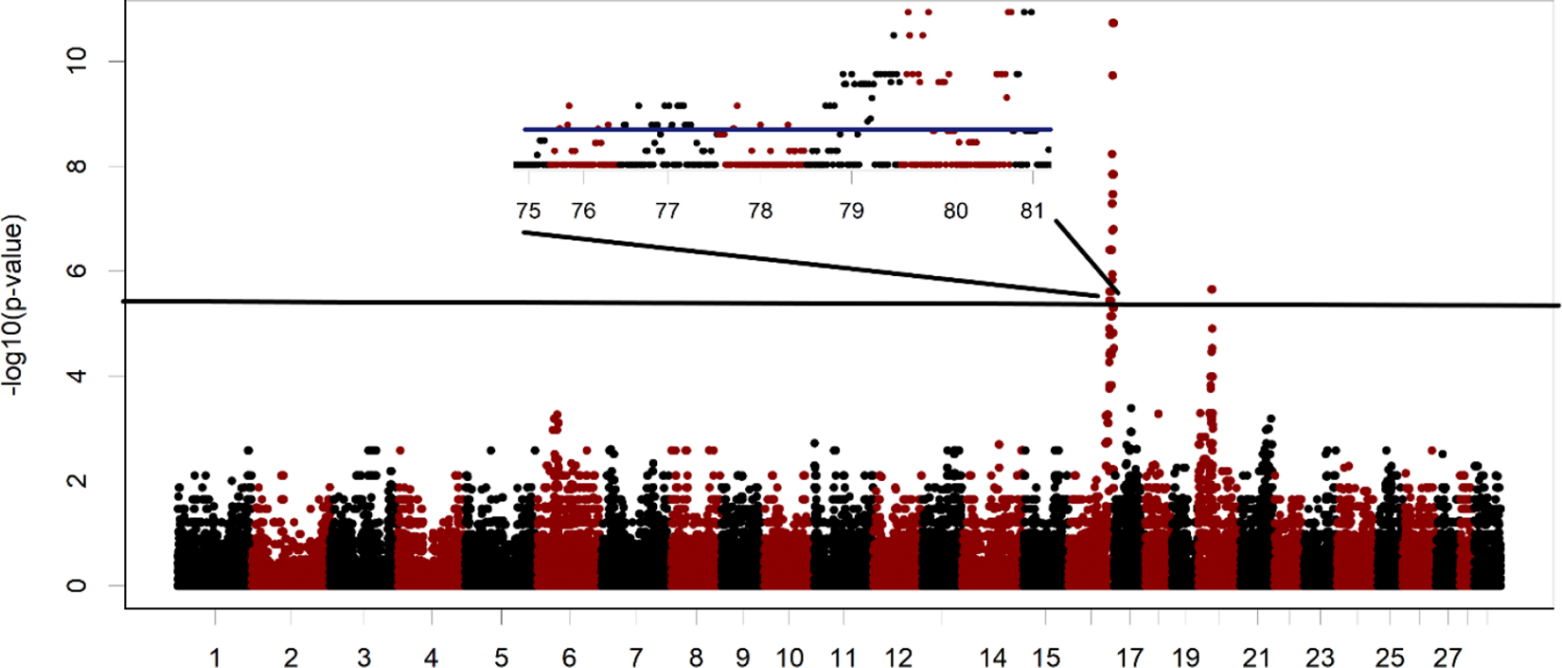
Manhattan plot of significance levels for association of recumbency with 101,917 DNA markers based on a recessive model with a significant region on chromosome 16 magnified; markers that exceed the genome-wide false discovery rate significance level are above the solid black (main) and blue (inset) lines.

**Table 2:** Genotypes of affected calves and control family members for a recumbency haplotype (RH) on chromosome 16

Item	Affected	Control
RH/RH	18	1
RH/−	0	16
−/−	0	9

A second region ranging from 9,585,914 to 10,754,653 bp that contained 3 significant markers was present on chromosome 20 with the recessive inheritance model (Figure 1), but the markers were not significantly associated according to the allelic or TDT models. Seventeen of 18 affected calves were homozygous for the markers and the remaining affected calf was heterozygous. Of the controls, 6 were homozygous for the same allele, 18 were heterozygous, and 2 were homozygous for the alternate allele. Whether this indicates that the condition involves multiple loci or was a spurious result is not certain. In contrast to observations on chromosome 16, the affected calves were not all homozygous for the remaining markers spanning the region.

Thirty-three of the affected calves had a known sire and were included in a pedigree analysis. Two generations of sire identification (sire and maternal grand-sire) were available for 9 calves from farm 1, whereas a minimum of 6 generations (calf's sire plus sires for 5 generations of dams) was available for all remaining calves. All known ancestors were traced separately for the sire and dam of each affected calf, and the number of calves where an ancestor was present in the sire and dam lineage was determined to identify plausible common ancestors.

The 33 affected calves with sire identification and all 24 affected calves with at least 5 generations of known maternal sires were traced to a bull born in 2008 who was considered a likely carrier ancestor. One prolific son of that bull was born in 2010 and was present in 30 of 33 paternal lineages and 23 of 24 maternal lineages. There were 66 daughters of the son with genotypes (48K to 77K) available from farm 3, the Pennsylvania State University dairy herd, and 3 commercial herds. Semen was obtained from the son to facilitate genotyping using the same chip (140K; BOVUHDV03, Neogen) that was used for the other animals in this study. The son's haplotype was determined using findhap.f90 based on his genotype and those of his daughters which confirmed that the son was a carrier of the suspect haplotype.

Calves homozygous for a haplotype on chromosome 16 had elevated risk of recumbency. While some calves partly recovered, the condition of most deteriorated and they were euthanized, often from development of secondary conditions related to weakness, including pneumonia. Curiously, the calves appeared healthy otherwise, maintaining good appetites and body condition.

Despite strong evidence for the candidate region, there is a degree of uncertainty due to the inconsistent development of the recumbency phenotype, existence of a homozygous calf that was able to stand without assistance, and an additional region with significant markers; this raises the possibility that the identified genomic region is spurious and that the condition is nongenetic. Evidence for the region harboring a recessive allele was supported by high statistical significance, the presence of heterozygous haplotypes in genotyped parents, a shared homozygous haplotype across multiple affected families, and a plausible path of inheritance.

Observations in humans and animal models indicate that genetic defects can result in recumbency at different ages. Most neuromuscular disorders have a genetic origin and mutations in a wide variety of genes can cause disease (Laing, 2012). Genetic recessive conditions and dominant de novo mutations have been associated with genetic pediatric neuromuscular disease in humans (Herman et al., 2021). A heritable juvenile-onset motor polyneuropathy was recently described in cats (Crawford et al., 2020).

Most of the calves in this study resulted from in vitro fertilization (**IVF**) and embryo transfer. Epigenetic aberrations and developmental abnormalities including large offspring syndrome have been associated with IVF in multiple species (Ventura-Juncá et al., 2015); however, the recumbent calves weighed 2.4 kg less than their normal counterparts on farm 3, which indicates that large offspring syndrome was not responsible. Moreover, 11 calves were affected that resulted from AI and 1 resulted from natural mating. That observation coupled with strong a genomic association suggests that IVF is not the determining factor for development of recumbency. Nevertheless, a genotype × environment interaction with IVF increasing the likelihood that the phenotype will be expressed when the genetic defect is inherited cannot be discounted.

The rate of inbreeding on both an annual and generational basis increased considerably following the introduction of genomic selection (Dechow et al., 2020; Makanjuola et al., 2020), which has implications for long-term selection response and increases the potential for genetic recessives to emerge (Fritz et al., 2013), or for low frequency mutations to amplify rapidly. For example, a neuropathy associated with splayed forelimbs in Jersey cattle has been recently identified that has increased in frequency from <1% in 2009 to over 8% currently (Al-Khudhair et al., 2022).

Genomic testing has accelerated discovery of genetic recessives that result in embryonic mortality (VanRaden et al., 2011b). Unfortunately, the haplotype screening method is not likely to identify conditions such as recumbency because calves may appear normal at birth and be genotyped as part of a normal management routine, and because the condition may not be completely penetrant. Genomic selection for heifer livability was introduced in national genetic selection programs in 2020 (Neupane et al., 2021); however, the heritability is low (<1%), resulting in modest levels of accuracy for genomic predictions. Moreover, heifer death results from a wide range of disease and injuries and it is not clear that genomic evaluation of a general quantitative heifer mortality trait would identify genetic recessives like that observed here unless a large proportion of calves were affected with the specific condition.

Current rates of inbreeding in the Holstein breed are favorable for rapid amplification of genetic defects. Support for a genetic defect on chromosome 16 that confers recumbency includes genome-wide significance that was shared across multiple mating combinations, heterozygous parent haplotypes, a plausible common ancestor for the affected pedigrees, and similar inherited disorders in other species. Despite strong evidence for the existence of a genetic condition, some caution is yet warranted because of the inconsistent phenotypic presentation of the calves and relatively small sample size. Future efforts to identify the exact location and type of mutation are needed to confirm the condition and facilitate genetic testing and selection.
